# Closing the loop in healthcare: chemically recycled ABS for 3D printing of medical device

**DOI:** 10.3389/fbioe.2026.1994426

**Published:** 2026-10-28

**Authors:** Alberto Bevilacqua, Davide Somma, Federica Potere, Simone Micalizzi, Nada Khaled Mansour, Andrea Rescalli, Cecilia Cappelli, Chiara Mirabella, Alessandra Simonelli, Andrea La Piccirella, Paolo Oliva

**Affiliations:** 1 IRCCS Humanitas Research Hospital, Rozzano, Lombardy, Italy; 2 Department of Biomedical Sciences, Humanitas University, Milan, Lombardy, Italy; 3 Versalis S.p.A., Viale Alcide De Gasperi, San Donato Milanese (MI), Lombardy, Italy

**Keywords:** chemical recycling, circular economy, non-implantable medical devices, point-of-care manufacturing, recycled ABS

## Abstract

**Introduction:**

The integration of three-dimensional (3D) printing into Point‐of‐Care (POC) healthcare has enabled the rapid production of patient-specific medical devices, including anatomical models and surgical guides. However, the increasing use of additive manufacturing in clinical settings raises environmental concerns related to the consumption of virgin polymers and the generation of plastic waste. Circular economy approaches based on recycled materials may represent a more sustainable alternative, provided that the safety, biocompatibility, and manufacturing requirements of medical applications are preserved. This study investigated the feasibility of using chemically recycled acrylonitrile butadiene styrene (ABS) for the fabrication of non‐implantable medical devices by Fused Deposition Modeling (FDM).

**Methods:**

The chemically recycled ABS used in this study was produced using Versalis’ proprietary technology. The recycled ABS was first chemically characterized to assess its suitability for biomedical use. It was then processed into 1.75 mm filaments and used to manufacture standardized specimens and proof‐of‐concept patient‐specific devices in an ISO 13485‐certified laboratory. Dimensional accuracy and process repeatability were evaluated, while biological performance was assessed through direct cytotoxicity testing according to ISO 10993‐5 using L929 fibroblasts. The results were compared with those obtained using a certified medical‐grade ABS reference material.

**Results:**

Chemically recycled ABS showed a chemical profile compatible with current regulatory requirements and was successfully processed into high‐quality FDM filaments and printed components. The printed specimens demonstrated high dimensional accuracy and reproducibility, supporting the suitability of the material for geometrically demanding medical applications. Cytotoxicity testing confirmed that the recycled ABS was non-cytotoxic in all tested forms, with 3D-printed samples showing a cytocompatibility profile comparable to that of the certified medical-grade ABS reference material. In addition, patient‐specific anatomical models and surgical guides were successfully fabricated, demonstrating the applicability of the material within clinically relevant POC workflows.

**Discussion:**

Overall, these findings indicate that chemically recycled ABS represents a promising and sustainable alternative to virgin polymers for the FDM manufacturing of non‐implantable medical devices. This study provides evidence that circular materials can be integrated into hospital‐based additive manufacturing without compromising the safety, biocompatibility, or manufacturing performance evaluated in this study, thereby supporting the transition toward more sustainable Point‐of‐Care production systems.

## 1 Introduction

The integration of Additive Manufacturing (AM) within clinical settings has experienced exponential growth, transitioning from a niche experimental tool to a foundational technology for modern healthcare (Ghosh et al., 2018). Point-of-Care (POC) 3D printing allows for the localized manufacturing of patient-specific anatomical models and surgical guides, shown to significantly improve clinical outcomes and reduce operative times (Ghosh et al., 2018; Mansour et al., 2025). However, this technological shift occurs within a sector facing severe environmental challenges (Ghosh et al., 2018). The healthcare industry is a significant consumer of resources and a major producer of emissions; average hospitals produce approximately 0.5 kg of waste per bed per day (Priyadarshini et al., 2024). Currently, the industry relies heavily on a linear “take-make-dispose” model, with plastics accounting for approximately 35% of all healthcare-related waste globally (Singh, Ogunseitan, and Tang, 2022). This represents a critical opportunity for the adoption of Circular Economy (CE) models that maximize material value while minimizing waste and energy (Priyadarshini et al., 2024; Mansour et al., 2025).

AM is recognized as a sustainable production technique that minimizes resource wastage due to its additive nature and ability to create customized tools without traditional tooling systems (Priyadarshini et al., 2024). Implementing sustainable 3D printing practices by recycling plastic waste into functional filaments has demonstrated potential for significant economic benefits, with cost savings reaching up to 85%, while drastically reducing the carbon footprint of medical manufacturing (Mansour et al., 2025). Despite these advantages, the implementation of AM for CE goals in healthcare gives rise to paradoxical tensions. Specifically, a conflict exists between the desire to close the loop through recycling and the strict requirement for high-quality raw materials that meet medical standards (Priyadarshini et al., 2024). The healthcare industry must conform to rigorous regulatory requirements for sterility, precision, and material quality; any microscopic refuge for contaminants could compromise patient safety (Priyadarshini et al., 2024). Furthermore, standard mechanical recycling of additive manufacturing plastics often faces challenges regarding material degradation and potential contamination, complicating its integration into regulated biomedical environments (Cruz et al., 2020). While mechanical recycling strategies have been actively explored to recover post-industrial ABS blends from the automotive and WEEE (Waste Electrical and Electronic Equipment) sectors, they typically require complex mixture designs to mitigate inherent property degradation (Zanatta et al., 2024).

To resolve these tensions and overcome these structural limitations, a transition from standard mechanical recycling to more advanced chemical recycling is proposed. While mechanically recycled polymers can suffer from non-homogeneous quality, chemically recycled materials provide a profile exactly identical to virgin polymers, ensuring consistency and eliminating hazardous contamination.

The current study serves as a feasibility validation of the technical and biological performance of this chemically recycled material. By transforming recycled ABS pellets into custom 1.75 mm filaments, the project rigorously assesses dimensional stability and cytocompatibility post-sterilization. The ultimate goal is to foster concrete circular economy practices in the biomedical sector, ensuring that 3D-printed devices maintain biological safety and avoid cytotoxic degradation during the thermal stress of additive manufacturing (Burkhardt et al., 2022).

## 2 Materials and methods

The overall experimental workflow is summarized in Figure 1. Briefly, chemically recycled ABS pellets supplied by Versalis were processed into 1.75 mm filaments and subsequently used for FDM 3D printing. Standardized specimens were manufactured to assess process feasibility, dimensional accuracy, and printing reproducibility, while proof-of-concept patient-specific anatomical models and surgical guides were fabricated to evaluate the applicability of the material to clinically relevant Point-of-Care workflows. Finally, the biological response of the material was assessed through direct cytotoxicity testing according to ISO 10993-5 using L929 fibroblasts.

**FIGURE 1:**
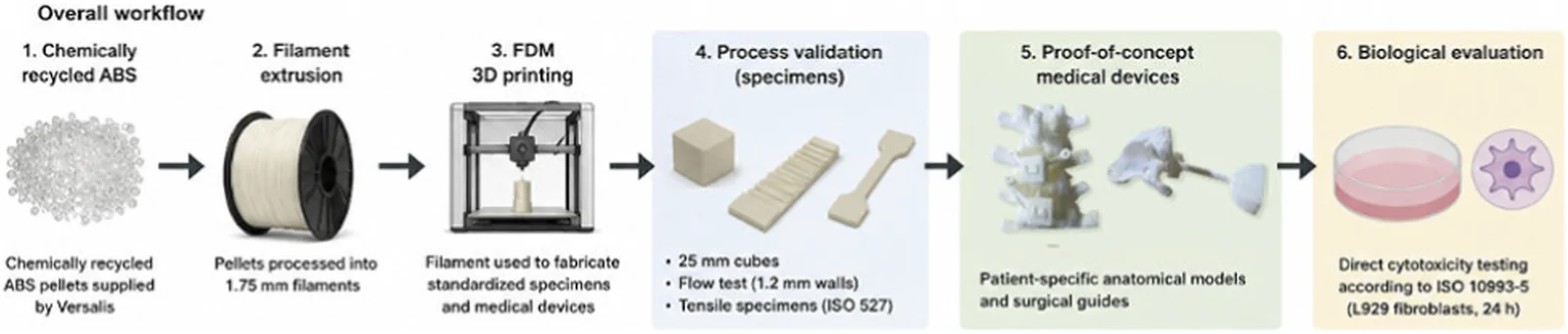
Overall workflow for the evaluation of chemically recycled ABS in Point-of-Care FDM manufacturing. Chemically recycled ABS pellets were processed into 1.75 mm filaments and used for FDM 3D printing of standardized process-validation specimens, including 25 mm cubes, flow-test specimens, and tensile specimens. The same material was then used to fabricate proof-of-concept patient-specific anatomical models and surgical guides. Biological evaluation was performed by direct cytotoxicity testing according to ISO 10993-5 using L929 fibroblasts after 24 h of exposure.

### 2.1 Chemical recycling of ABS

Chemical recycling has emerged as a complementary strategy to mechanical recycling for plastic waste streams that are heterogeneous, contaminated, or unsuitable for direct mechanical reprocessing (Jeswani et al., 2021; Kumagai et al., 2025). Unlike mechanical recycling, which preserves the polymer structure but may be associated with progressive material degradation and variability in composition, chemical recycling converts plastic waste into molecular feedstocks that can be reintroduced into conventional petrochemical production processes (Jeswani et al., 2021; Kumagai et al., 2025).

In this study, chemically recycled ABS supplied by Versalis was selected as the target material for Point-of-Care additive manufacturing. The recycled feedstock was obtained through a certified chemical recycling pathway based on pyrolysis-derived circular feedstocks and allocated according to a mass-balance approach. Under this framework, recycled inputs are tracked through a verified accounting system, allowing a corresponding share of recycled content to be attributed to the final polymer product. In this way, the recycled content is attributed using standardized and certified criteria (ISCC International Sustainability and Carbon Certification, 2024).

The use of chemically recycled feedstocks is aligned with the objectives of the European Strategy for Plastics in a Circular Economy and subsequent Circular Economy Action Plans, which promote increased incorporation of recycled raw materials while maintaining product performance and safety requirements (“European Commission - European Commission - Plastics Strategy, 2018).

This approach was considered particularly relevant for biomedical manufacturing because it may reduce the variability and contamination risks associated with heterogeneous mechanically recycled polymer streams, while enabling the production of ABS grades designed to meet the same technical specifications as conventional virgin counterparts. Therefore, chemically recycled ABS was investigated as a candidate circular material for the fabrication of non-implantable medical devices by FDM, with subsequent evaluation of printability, dimensional accuracy, and *in vitro* cytocompatibility.

### 2.2 Chemical and normative screening

Chemical and regulatory screening was initially performed on mechanically recycled ABS streams supplied by Versalis. Given that plastics recovered from Waste Electrical and Electronic Equipment (WEEE) may contain legacy additives, including brominated flame retardants and heavy metals (European Parliament and Council of the European Parliamentand Council of the European Union, 2006), the screening aimed to assess hazardous substances and non-intentionally added substances (NIAS) potentially relevant to biomedical applications. The analyses included thermogravimetric analysis (TGA) for ash residue and filler content, targeted quantification of halogens and trace metals, and screening for secondary polymers, residual monomers, organic additives, and volatile compounds.

The resulting chemical profile was assessed against relevant regulatory frameworks, including Annex XVII of the REACH Regulation (European Parliament and Council of the European Parliamentand Council of the European Union, 2006), the RoHS Directive (“Directive 2011/65/EU of the European Parliament and of the Council on the Restriction of the Use of Certain Hazardous Substances in The European Parliament And The Council Of The European Union, 2011), and IEC 61249-2-21 for halogen restrictions (International Organization for Standardization 2016). Although no prohibited hazardous substances were identified in the mechanically recycled ABS streams, chemically recycled ABS was selected for subsequent filament extrusion, FDM printing, and biological testing to reduce the potential variability and contamination risks associated with heterogeneous mechanical recycling streams.

### 2.3 Filament extrusion and 3D printing setup

Following the chemical validation, the physical transformation of the recycled ABS pellets into printable material took place. Pre-drying conditions, including specific temperature and duration parameters, were evaluated and applied to the industrial recycled pellets. The extrusion process was subsequently configured to target a precise 1.75 mm filament diameter using the 3Devo Filament Maker ONE (3devo B.V., Utrecht, Netherlands). All 3D printing operations were conducted within an ISO 13485 certified university-based lab (Buekens and Yang, 2014) to ensure compliance with medical manufacturing quality standards. To guarantee reproducibility and optimal material processing, specific Fused Deposition Modeling (FDM) printing parameters were established. The target operational ranges were carefully calibrated to ensure that the material’s inherent rheological and mechanical properties were not compromised during the thermal stress of manufacturing. Extrusion-based processing of ABS within inappropriate temperature windows is known to trigger structural polymer alteration and volatile thermal degradation by products (Wojtyła, Klama, and Baran, 2017). Therefore, maintaining strict control over the thermal profile was crucial to prevent material degradation and ensure safety for subsequent medical validation.

### 2.4 Process characterization: printability and geometric validation

Building upon the established extrusion and printing parameters, a testing framework was implemented to quantitatively characterize the printability, dimensional stability, and geometric fidelity of the material. Standardized test artifacts were fabricated to evaluate key aspects of the FDM process. These included a temperature tower to determine the optimal printing temperature range, three solid test cubes with a target CAD dimension of 25.00 mm to assess dimensional stability and repeatability across multiple axes, a flow-test specimen with a CAD target wall thickness of 1.20 mm, printed using a standard 0.4 mm nozzle, and ISO 527-2 dog-bone specimens to assess the reproducibility of geometrically standardized printed parts.

### 2.5 *In vitro* direct cytotoxicity

Direct cytotoxicity testing was carried out in accordance with ISO 10993-5 (International Organization for International Organization for Standardization, 2009). The assay was performed on both chemically recycled ABS materials supplied by Versalis and a certified medical-grade ABS filament provided by Innovatefil (Smart Materials 3D, Spain), which was used as reference control material due to its established medical-grade classification.

L929 was routinely cultured in complete culture medium composed of DMEM supplemented with 10% FBS and 1% penicillin/streptomycin antibiotics. Cells were seeded in 24-well plates at a density of 5x10^5^ cells per well and incubated at 37 °C in a humidified atmosphere containing 5% CO_2_, until reaching approximately 70% confluency.

To evaluate whether the thermal processing associated with additive manufacturing affected the biological response of the material, different ABS material forms were tested (N = 3 for each material): raw ABS pellets (only from Versalis), extruded ABS filament, and 3D-printed ABS cylinders. The 3D-printed specimens were designed to cover approximately 10% of the well surface area, in accordance with ISO 10993-5.

As negative control, three wells containing only L929 cells cultured in complete medium, without any material exposure, were included in the experimental setup.

Prior to cell exposure, all material samples were sterilized using vaporized hydrogen peroxide, following a standard workflow commonly applied for medical device sterilization. Cells were then exposed to the tested materials under standard culture conditions for 24 h, after which cell viability was assessed. After 24 h, alamarBlue™ assay was performed according to the manufacturer’s instructions. Absorbance at 570 nm and 600 nm was measured by using a microplate reader (infinite 200Pro, Tecan) and percentage viability was computed.

Each experimental condition was evaluated in three independent experiments, each performed in technical triplicate. For each experiment, technical triplicates were averaged before statistical analysis, and the independent experiments were used as the statistical unit. Cell viability data are reported as mean ± standard deviation. Statistical analysis was performed using one-way analysis of variance (ANOVA) to compare cell viability among the tested material configurations. A p-value <0.05 was considered statistically significant.

### 2.6 Proof of concept: Clinical prototypes

Following the comprehensive technical and biological characterization of the chemically recycled ABS, a final proof of concept was conducted to validate the practical feasibility of the production process. Functional prototypes, specifically anatomical models and patient-specific surgical cutting guides were 3D printed to verify the material’s performance and geometric precision when applied to complex, real-world medical device designs.

## 3 Results

### 3.1 Chemical-physical characteristics, sustainability, and normative screening

The initial analytical evaluation of the mechanically recycled ABS streams, sourced from Waste Electrical and Electronic Equipment (WEEE) and the automotive sector, yielded crucial data regarding the material’s baseline safety. The comprehensive chemical screening successfully confirmed the absence of prohibited hazardous substances within the supplied materials. The results of the halogen, filler, and trace-metal analyses are summarized in Table 1, while the secondary polymers and non‐intentionally added substances (NIAS) identified in the recycled ABS streams are reported in Table 2.

**TABLE 1:** Chemical screening: Halogens, fillers, and trace metals analysis of recycled ABS. *Dl = Detection limit (5 ppm).

Halogens	​	R-ABS from WEEE	R-ABS from automotive
Chlorine	ppm	100-400	20-200
Bromine	ppm	< Dl*	10-300
Fluorine	ppm	< Dl*	< Dl*
Iodine	ppm	< Dl*	< Dl*
FILLERS	​	​	​
TGA residue at 800 °C	%	2%-8%	2%-3%
CaCO_3_	%	Max 2%	Max 1%
Non-CaCO_3_ filler	%	2%-6%	0.1%-2%
Total carbon	%	Max 2%	Max 3% (depending on color)
Ash (including CaCO_3_)	%	2%-7%	Max 3%
METALS	​	​	​
Al	ppm	300-1500	Max 200
As	ppm	Absent	Absent
Ba	ppm	Not analyzed	10-150
Ca	ppm	500-8000	50-5000
Cd	ppm	<0.1	<0.1
Co.	ppm	Absent	Absent
Cr	ppm	<10	<10
Cu	ppm	<20	<25
Fe	ppm	10-200	10-200
K	ppm	10-100	10-200
Mg	ppm	<300	<400
Mn	ppm	Absent	Absent
Mo	ppm	Not analyzed	<20
Na	ppm	20-100	20-150
Ni	ppm	Absent	<20
Pb	ppm	Absent	<15
Sb	ppm	Absent	<50
Se	ppm	Absent	<20
Si	ppm	Not analyzed	<250
Sn	ppm	Absent	<100
Ti	%	<4%	<2%
Tl	ppm	Absent	<10
V	ppm	Absent	Absent
Zn	ppm	10-200	<100
Hg	ppm	<1	<1

**TABLE 2:** Secondary polymers and non-intentionally added substances (NIAS) profile.

Other polymers	r-ABS from WEEE	r-ABS from automotive	Notes
PMMA	Traces	​	​
PC	Traces	​	​
Other substances			
Styrene	Present	Present	Typical of ABS
4-Vinylcyclohexene	Present	Present	Typical of ABS
Isobutene	Present	Present	Typical of ABS
Methyl acrylate, methyl methacrylate	Present	Present	Typical of ABS
Toluene	Present	Present	Typical of ABS
Ethylbenzene	Present	Present	Typical of ABS
Cumene	Present	Present	Typical of ABS
a-methylstyrene	Present	Present	Typical of ABS
Acetaldehyde	Traces	Traces	Typical of ABS
Isopentane	Traces	Traces	Typical of ABS
Acrylonitrile	Traces	Traces	Typical of ABS
Benzene	Traces	Traces	Typical of ABS
n-propylbenzene	Traces	Traces	Typical of ABS
Other additives (added in application formulations)			
Oil	Traces	Traces	​
Wax	Traces	Traces	​
Irganox 1076	Present	Present	​
Tinuvin 770	Present	Present	​
Santowhite, also known as sumilizer BBM (CAS no. 85-60-9)	Present	Present	​
Dehydroabietic acid	Present	Present	​
Octyl palmitate and octyl stearate, palmitic acid and stearic acid, stearyl alcohol	Present	Present	​
Bisphenols	Present	Present	​
Ethylene bis-stearamide (probable)	Present	Present	​

Importantly, although the mechanically recycled ABS streams did not show prohibited substances in the preliminary screening, chemically recycled ABS was selected for the subsequent experimental validation because this route is expected to provide a more controlled and reproducible feedstock, with technical specifications comparable to those of conventional virgin ABS grades and a reduced risk of contamination associated with heterogeneous mechanical recycling streams.

### 3.2 Filament production and 3D printing validation of virgin materials

Following the preliminary benchmarking phase, four virgin ABS formulations—Impressio ABS D11, Impressio ABS B10, Impressio ABS C02, and Impressio ABS C06 P—were evaluated to identify the most suitable grade for Fused Deposition Modeling (FDM) before transferring the process to the chemically recycled counterpart. Among the tested formulations, the selected grade showed the most favorable printing behavior, characterized by improved layer adhesion, limited thermal warping, and good surface quality (Figure 2).

**FIGURE 2:**
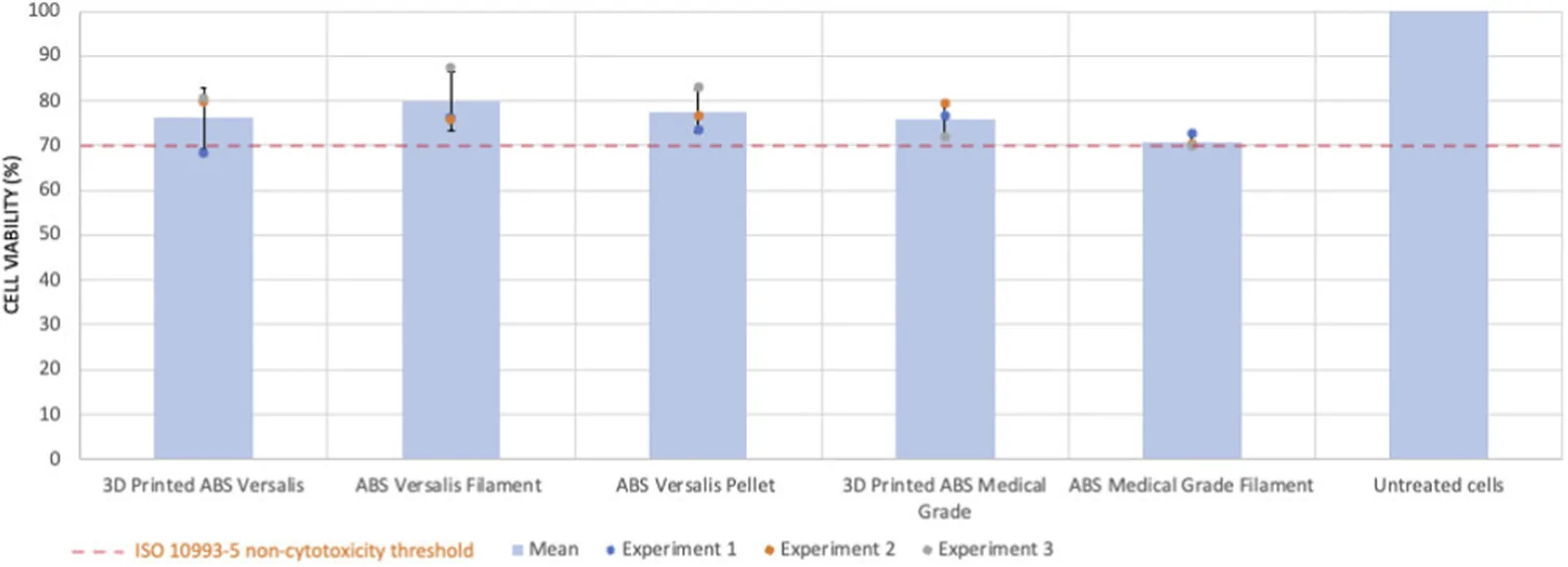
Cell viability of Versalis chemically recycled ABS and medical-grade ABS reference materials after 24 h of exposure. Bars represent mean cell viability values, while individual dots indicate independent experimental replicates. The dashed line indicates the ISO 10993‐5 non‐cytotoxicity threshold, corresponding to 70% cell viability relative to the negative control. Untreated cells were used as control and set at 100% viability.

Printability and dimensional stability were further assessed by fabricating standardized ISO/FDIS 527-2 dog-bone specimens, solid cubes with a target dimension of 25.00 mm, and flow-test specimens with a target wall thickness of 1.20 mm for each formulation (Figure 2). Detailed extrusion and printing parameters, together with the complete geometric validation datasets for the four virgin ABS formulations, including dog-bone specimens, solid cubes, flow tests, and temperature towers, are reported in the Supplementary Material (Supporting Information, Supplementary Sections S1, S2).

Representative standardized specimens fabricated during the preliminary FDM benchmarking phase are shown in Figure 3.

**FIGURE 3:**
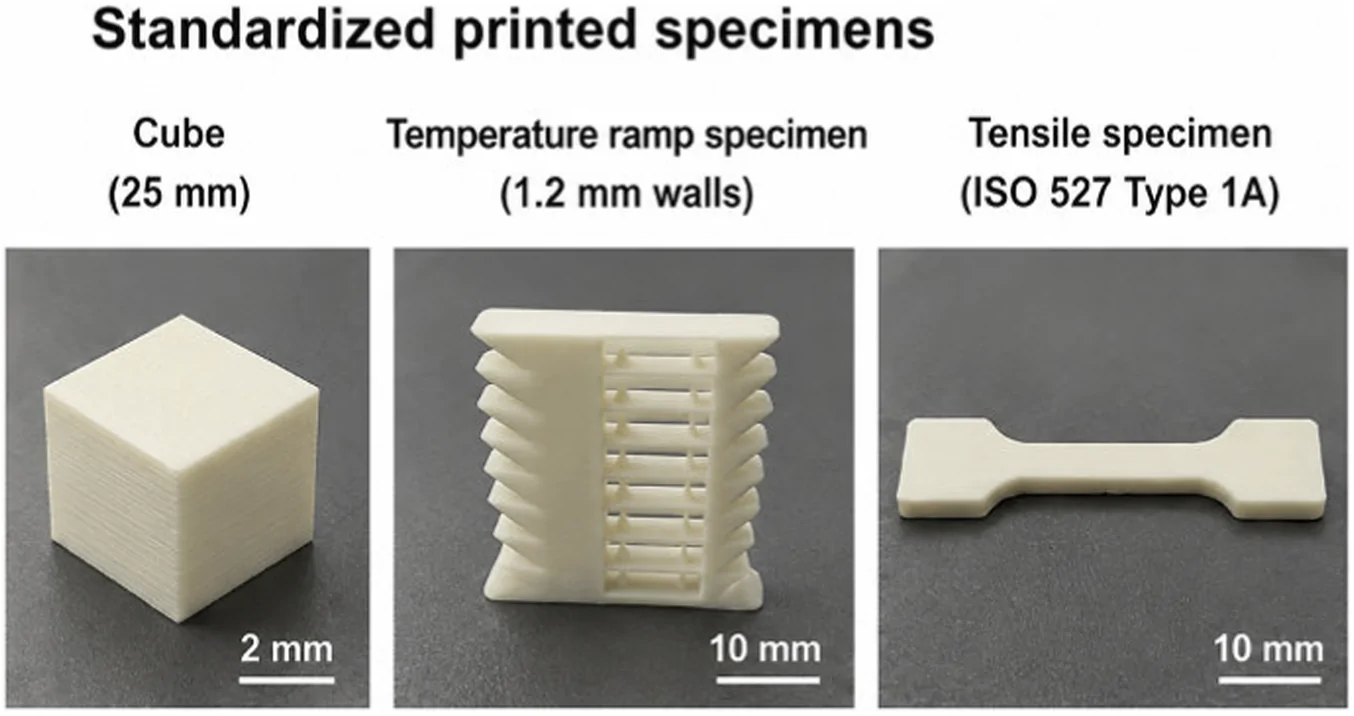
Representative standardized specimens fabricated for FDM process benchmarking and validation. The printed geometries included a solid cube with a target dimension of 25 mm, temperature ramp specimen with 1.2 mm target wall thickness, and an ISO 527 Type 1A tensile specimen. These specimens were used to assess printability, dimensional stability, extrusion consistency, and geometric reproducibility during process optimization. Scale bars are shown in each panel.

### 3.3 Printability and geometric validation of chemically recycled ABS

Building upon the foundational parameters established with the virgin formulations, the physical transformation of the chemically recycled ABS pellets into a continuous 1.75 mm filament was achieved consistently.

The optimal extrusion and printing parameters defined for the industrialized chemically recycled ABS filament are detailed in Supplementary Sections S3 of the Supplementary Material (Supporting Information, Supplementary Table S5).

Subsequent 3D printing tests demonstrated that the chemically recycled material possessed excellent geometric fidelity, which is a critical, non-negotiable requirement for manufacturing precise surgical instruments such as pedicle screw guides.

Quantitative dimensional analysis conducted on specific test geometries reinforced these findings. The 3D printed solid cubes achieved an average dimension of 24.93 mm against a CAD target of 25.00 mm, with a remarkably low standard deviation of 0.02 mm. This indicates highly reliable and repeatable dimensional stability with minimal volumetric shrinkage. Furthermore, the flow test revealed a measured average thickness of 1.22 mm with a standard deviation of 0.01 mm, confirming excellent rheological control during the material deposition phase. Finally, the standardized ISO 527 Type 1A and Type 1B specimens exhibited average percentage deviations that remained consistently below 1.5% across the primary mechanical axes.

The complete photographic report and quantitative dimensional measurements for the 3D-printed recycled ABS samples, including ISO 527 specimens, test cubes, cutting guides, and vertebral guides, are comprehensively documented in the Supplementary Material (Supporting Information, Supplementary Sections S3; Supplementary Figures S13–S14).

### 3.4 Chemical sterilization process and biological compatibility

Cytotoxicity testing was performed to assess the biological response of Versalis chemically recycled ABS in comparison with a certified medical-grade ABS reference material (Figure 3). According to ISO 10993-5, materials are considered non-cytotoxic when cell viability remains above 70% relative to the negative control. All tested configurations of Versalis chemically recycled ABS showed cell viability values above this threshold, indicating the absence of cytotoxic effects under the investigated conditions (Figure 4).

**FIGURE 4:**
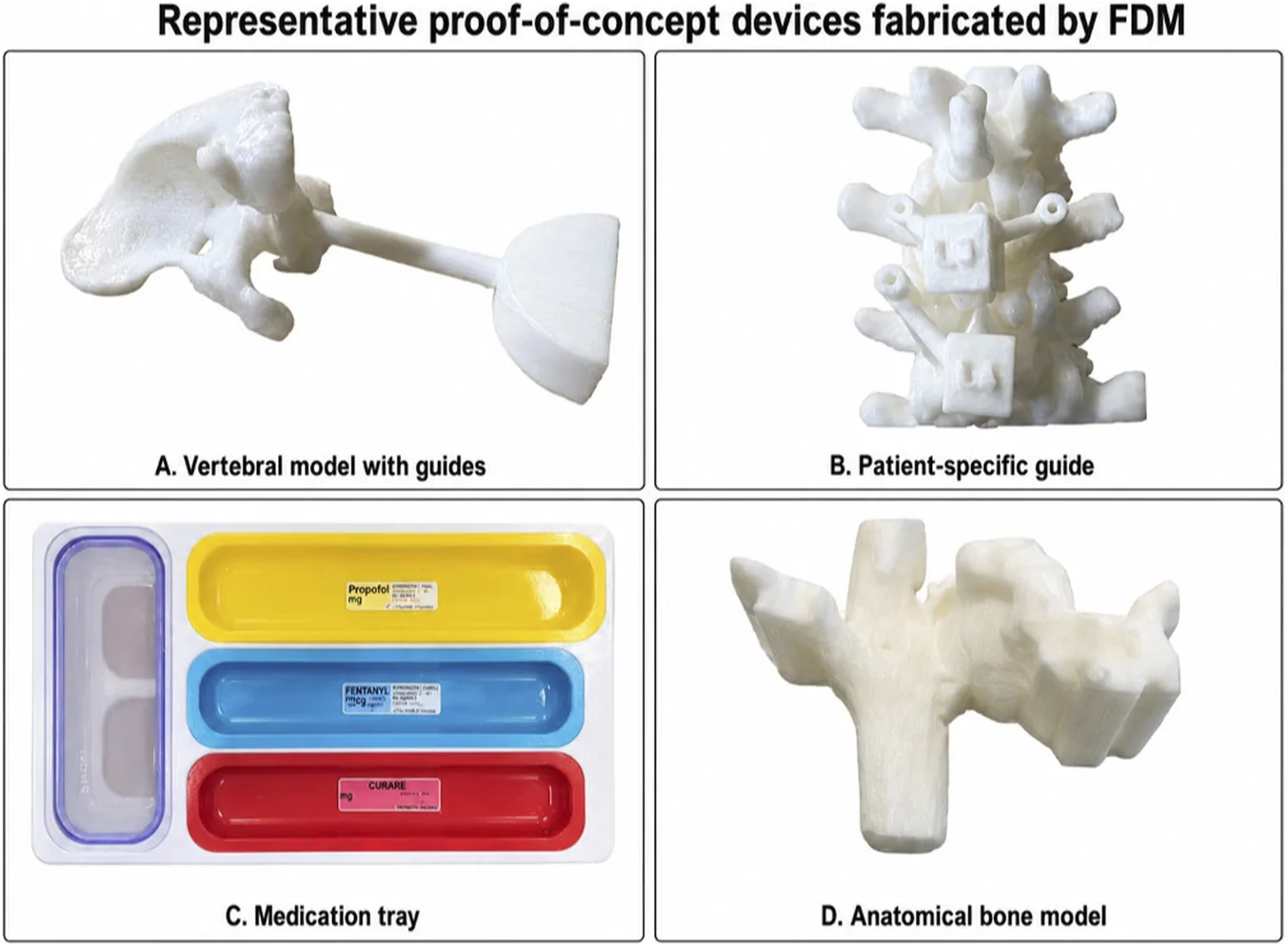
Representative proof‐of‐concept devices fabricated by FDM using chemically recycled ABS. Examples of printed prototypes include patient‐specific anatomical models, surgical guides **(A,D)**, a vertebral model with guides **(B)**, and a medication tray **(C)**. These devices were fabricated to assess the feasibility of processing chemically recycled ABS into complex geometries relevant to Point‐of‐Care medical manufacturing.

In particular, cell viability was 77.52% for raw Versalis ABS pellets, 79.73% for Versalis ABS filament, and 76.10% for 3D-printed Versalis ABS samples. These results indicate that the chemically recycled ABS maintained a non-cytotoxic profile after both filament extrusion and additive manufacturing, suggesting that the thermal processing steps did not negatively affect the cytocompatibility of the material.

When compared with the certified medical-grade ABS reference, 3D-printed Versalis ABS showed a comparable cytocompatibility profile. Cell viability was 76.10% for 3D-printed Versalis ABS and 75.84% for 3D-printed medical-grade ABS. A similar trend was observed for the filament samples, with both Versalis ABS and medical-grade ABS remaining above the ISO 10993-5 non-cytotoxicity threshold, showing viability values of 79.73% and 70.75%, respectively. Notably, while the medical-grade ABS filament showed a viability value close to the acceptance threshold, Versalis ABS filament maintained a higher cell viability.

Overall, Versalis chemically recycled ABS was non-cytotoxic in all tested configurations. Importantly, the 3D-printing process did not reduce cell viability below the ISO 10993-5 acceptance threshold, indicating that additive manufacturing did not compromise the cytocompatibility of the chemically recycled ABS under the experimental conditions adopted in this study.

### 3.5 Proof-of-concept fabrication of patient-specific prototypes

To assess the practical applicability of chemically recycled ABS in clinically relevant Point-of-Care workflows, proof-of-concept patient-specific prototypes were fabricated by FDM. The material was successfully used to print representative anatomical models, surgical guides, and other non-implantable prototypes with complex geometries. Visual inspection confirmed complete fabrication of the intended designs, with adequate surface quality and no evident macroscopic defects, delamination, or severe warping. Representative proof-of-concept devices are shown in Figure 5. These findings support the feasibility of using chemically recycled ABS for the production of customized non-implantable medical devices and accessories through Point-of-Care additive manufacturing.

**FIGURE 5:**
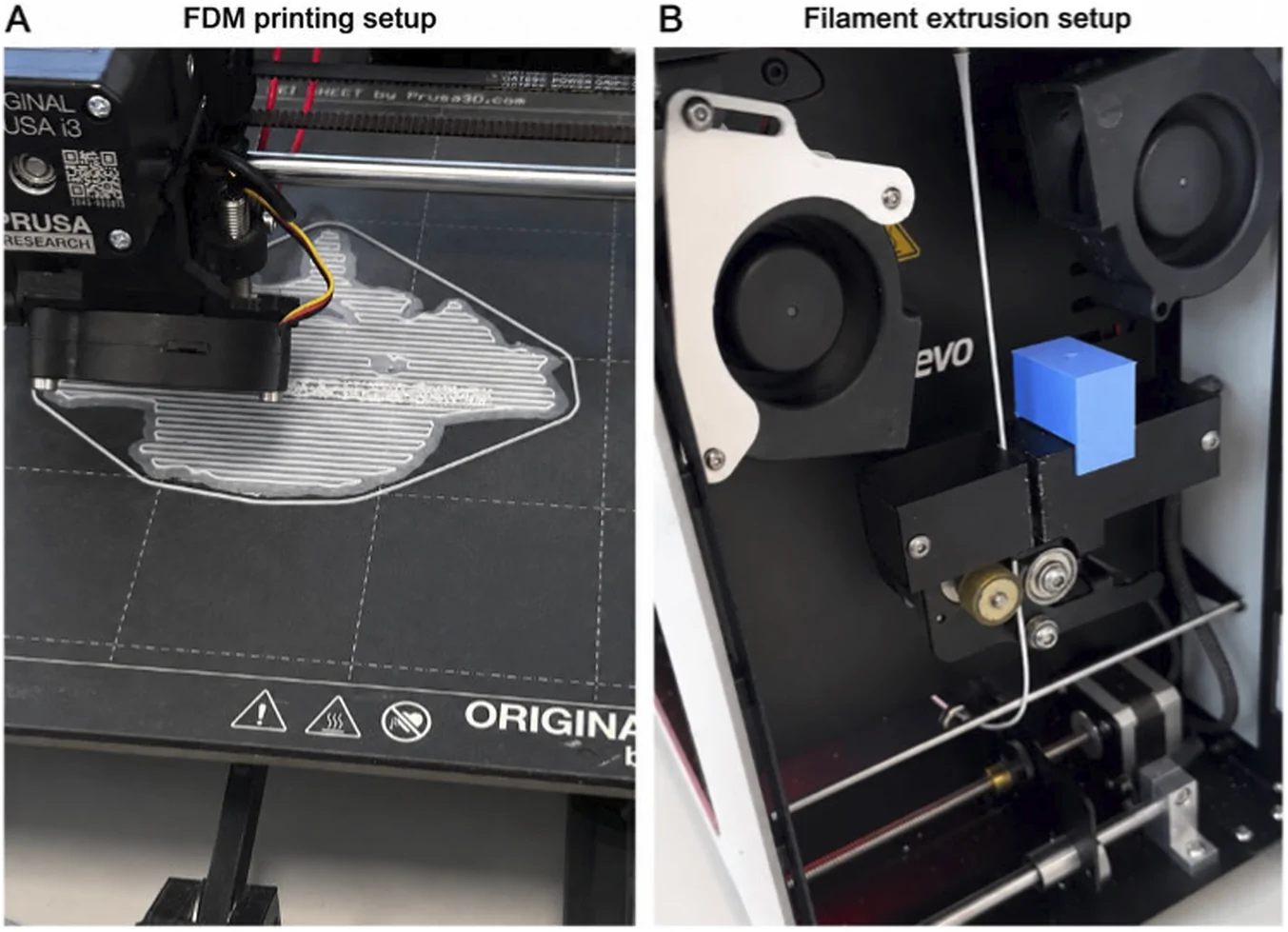
Experimental setup used for filament extrusion and FDM printing. **(A)** Representative FDM printing setup used for the fabrication of test specimens and proof‐of‐concept devices. **(B)** Filament extrusion setup used to produce 1.75 mm filament from the selected ABS formulation prior to FDM processing.

## 4 Discussion

The present study provides preliminary evidence on the potential use of chemically recycled ABS as a feedstock for biomedical additive manufacturing, supporting the integration of circular economy principles into medical manufacturing workflows. Chemically recycled ABS pellets supplied by Versalis were successfully processed into filament and subsequently used to fabricate 3D-printed specimens with adequate dimensional stability. This approach is particularly relevant in the context of customized medical manufacturing, where additive manufacturing enables the production of patient-specific models and devices while potentially reducing the environmental impact associated with polymer-based materials. One of the main challenges in the use of recycled polymers for biomedical applications is the ability to preserve dimensional accuracy and reproducibility after processing. In this study, the chemically recycled ABS showed an average dimensional deviation below 1.5% along the main axes, suggesting that the recycling, extrusion, and printing steps did not markedly affect the geometrical fidelity of the printed samples. In addition to dimensional performance, biological safety represents a critical requirement for any material intended for clinical use. Direct cytotoxicity testing showed that Versalis chemically recycled ABS was non-cytotoxic in all tested configurations, including raw pellets, extruded filament, and 3D-printed samples, with cell viability values above the 70% threshold defined by ISO 10993-5. Specifically, cell viability was 76.10% for 3D-printed Versalis ABS, 79.73% for Versalis ABS filament, and 77.52% for raw pellets. These findings suggest that the thermal and mechanical stresses associated with filament extrusion and fused filament fabrication did not induce a cytotoxic response under the tested conditions. When compared with the certified medical-grade ABS reference, the 3D-printed chemically recycled ABS showed a comparable biological response, with no significant difference from the 3D-printed medical-grade ABS, which reached 75.84% cell viability. Overall, the results indicate that chemically recycled ABS may represent a promising material for selected non-implantable medical applications produced by additive manufacturing. The in-hospital fabrication of patient-specific anatomical models and surgical tools represents a rapidly expanding paradigm in Point-of-Care healthcare, where material safety and regulatory compliance remain paramount (International Organization for International Organization for Standardization, 2009; Chan, Tan, and Wu, 2017). By demonstrating both biocompatibility and structural integrity, this upcycled ABS grade fully aligns with the strict requirements of hospital-based manufacturing workflows.

## Data Availability

The raw data supporting the conclusions of this article will be made available by the authors, without undue reservation.
