# Supplementary material for: Closing the loop in healthcare: chemically recycled ABS for 3D printing of medical device
Source: Front Bioeng Biotechnol. 2026 Oct 28;14:1994426. doi: 10.3389/fbioe.2026.1994426 (PMC13751352; doi:10.3389/fbioe.2026.1994426)
Supplement: Supplementary file 1 [file DataSheet1.docx]

**Supporting Information**

**Closing the Loop in Healthcare: Chemically Recycled ABS for 3D Printing of Medical Device**

**Section S1: Extrusion and 3D Printing Parameters**

Following the preliminary benchmarking phase, the evaluation focused on the four distinct virgin ABS formulations previously specified (Impressio ABS D11, Impressio ABS B10, Impression ABS C02, and Impressio ABS C06 P). The objective was to identify the most suitable grade for Fused Deposition Modeling (FDM) prior to industrializing the recycled counterpart.

Tab. S1. Impressio ABS D11 Extrusion and Printing Parameters

| **PELLET PRE-DRYING** | |
| --- | --- |
| Temperature | 80°C for 2-4 hours |
| **FILAMENT EXTRUSION PARAMETERS** | |
| Temperature (°C) | 210 – 225 – 230 - 240 |
| Extrusion speed (rpm) | 4 |
| Filament thickness (mm) | 1,75 ± 0,005 |
| Cooling fan speed (%) | 15 |
| **PRINTING PARAMETERS** | |
| Nozzle temperature | 225 ± 260 |
| Bed temperature | 90 ± 110 |
| Flow | 98 ± 105 |
| Layer thickness (mm) | 0.2 |
| Printing speed (mm/s) | 30 ± 50 |
| Retraction (mm) | 0.8 ± 1.2 |

Tab. S2. Impressio ABS B10 Extrusion and Printing Parameters

| **PELLET PRE-DRYING** | |
| --- | --- |
| Temperature | 80°C for 2-4 hours |
| **FILAMENT EXTRUSION PARAMETERS** | |
| Temperature (°C) | 210 – 225 – 230 - 240 |
| Extrusion speed (rpm) | 4 |
| Filament thickness (mm) | 1,75 ± 0,005 |
| Cooling fan speed (%) | 15 |
| **PRINTING PARAMETERS** | |
| Nozzle temperature | 230 ± 260 |
| Bed temperature | 90 ± 110 |
| Flow | 98 ± 105 |
| Layer thickness (mm) | 0.2 |
| Printing speed (mm/s) | 30 ± 50 |
| Retraction (mm) | 0.8 ± 1.2 |

Tab. S3. Impressio ABS C02 Extrusion and Printing Parameters

| **PELLET PRE-DRYING** | |
| --- | --- |
| Temperature | 80°C for 2-4 hours |
| **FILAMENT EXTRUSION PARAMETERS** | |
| Temperature (°C) | 210 – 225 – 230 - 240 |
| Extrusion speed (rpm) | 4 |
| Filament thickness (mm) | 1,75 ± 0,005 |
| Cooling fan speed (%) | 15 |
| **PRINTING PARAMETERS** | |
| Nozzle temperature | 235 ± 260 |
| Bed temperature | 90 ± 110 |
| Flow | 98 ± 105 |
| Layer thickness (mm) | 0.2 |
| Printing speed (mm/s) | 30 ± 50 |
| Retraction (mm) | 0.8 ± 1.2 |

Tab. S4. Impressio ABS C06 P Extrusion and Printing Parameters

| **PELLET PRE-DRYING** | |
| --- | --- |
| Temperature | 80°C for 2-4 hours |
| **FILAMENT EXTRUSION PARAMETERS** | |
| Temperature (°C) | 210 – 225 – 230 - 240 |
| Extrusion speed (rpm) | 4 |
| Filament thickness (mm) | 1,75 ± 0,005 |
| Cooling fan speed (%) | 15 |
| **PRINTING PARAMETERS** | |
| Nozzle temperature | 225 ± 260 |
| Bed temperature | 90 ± 110 |
| Flow | 98 ± 105 |
| Layer thickness (mm) | 0.2 |
| Printing speed (mm/s) | 30 ± 50 |
| Retraction (mm) | 0.8 ± 1.2 |

**Section S2: Geometric and Mechanical Validation of Virgin ABS Formulations**

Guide to Reading the Geometric Validation Data Sheets (Dog-Bone Specimens):

The following figures and tables present a comprehensive geometric and dimensional validation workflow for the 3D-printed specimens (ISO 527 Dog-Bone Type 1A and 1B). Each data sheet is organized into four main diagnostic sections to evaluate manufacturing fidelity and alignment with original CAD specifications:

- **Specimen Schematics:** Technical drawing illustrating the nominal dimensions (in mm) and geometric tolerances of the standardized ISO 527 specimen used as the reference target for the 3D printing process.
- **3D Printing Parameters:** Table detailing the core slicing and manufacturing configurations utilized during the Fused Deposition Modeling (FDM) process.
- **Dimensional Measurements Matrix:** Quantitative dataset collected across three distinct printed samples (Sample 1, Sample 2, Sample 3). Measurements are reported for critical architectural features. Each parameter is benchmarked against the target CAD value to calculate the exact percentage deviation ($\Delta\%$), serving as an index of volumetric shrinkage or over-extrusion.
- **Statistical Summary and Real-World Visuals:** A summary matrix reporting the calculated Mean value, Standard Deviation (SD), and Average Percentage Deviation across the sampled cohort, paired with a photographic rendering of the actual 3D-printed specimens to visually assess physical integrity.

*Note for the reader: The supporting data sheets allow a direct correlation between the process parameters and the resulting geometric accuracy of the printed parts. A low standard deviation (SD) combined with an average percentage deviation close to zero indicates high process repeatability and optimal calibration of the material's extrusion multiplier, ensuring that the physical component faithfully reproduces the digital clinical model.*

1. Impressio ABS D11

The dimensional accuracy of the Impressio ABS D11 printed parts showed highly consistent results with minimal percentage deviation across multiple test geometries.

Fig. S1. Impressio ABS D11 Dog-Bone Type 1A

Fig. S2. Impressio ABS D11 Dog-Bone Type 1B

Fig. S3. Impressio ABS D11 Solid Cube, Flow Test, and Temp Tower

2. Impressio ABS B10

The physical testing for the Impressio ABS B10 formulation demonstrated excellent repeatability, maintaining tight tolerances against the original CAD dimensions.

Fig. S4. Impressio ABS B10 Dog-Bone Type 1A

Fig. S5. Impressio ABS B10 Dog-Bone Type 1B

Fig. S6. Impressio ABS B10 Solid Cube, Flow Test, and Temp Tower

3. Impressio ABS C02

Dimensional analysis for the Impressio ABS C02 printed specimens yielded the following standardized measurement data.

Fig. S7. Impressio ABS C02 Dog-Bone Type 1A

Fig. S8. Impressio ABS C02 Dog-Bone Type 1B

Fig. S9. Impressio ABS C02 Solid Cube, Flow Test, and Temp Tower

4. Impressio ABS C06 P

The final virgin formulation, Impressio ABS C06 P, underwent the same rigorous geometric validation, with the structural precision detailed below.

Fig. S10. Impressio ABS C06 P Dog-Bone Type 1A

Fig. S11. Impressio ABS C06 P Dog-Bone Type 1B

Fig. S12. Impressio ABS C06 P Solid Cube, Flow Test, and Temp Tower

**Section S3: Geometric and Mechanical Validation of Recycled ABS Final Product**

Building upon the foundational parameters established with the virgin formulations, the physical transformation of the chemically recycled ABS pellets into a continuous 1.75 mm filament was achieved consistently. Subsequent 3D printing tests demonstrated that the recycled material possessed excellent geometric fidelity, which is a critical, non-negotiable requirement for manufacturing precise surgical instruments such as pedicle screw guides.

Quantitative dimensional analysis conducted on specific test geometries reinforced these findings. The 3D printed solid cubes achieved an average dimension of 24.93 mm against a CAD target of 25.00 mm, with a remarkably low standard deviation of 0.02 mm. This indicates highly reliable and repeatable dimensional stability with minimal volumetric shrinkage. Furthermore, the flow test revealed a measured average thickness of 1.22 mm with a standard deviation of 0.01 mm, confirming excellent rheological control during the material deposition phase. Finally, the standardized ISO 527 Type 1A and Type 1B specimens exhibited average percentage deviations that remained consistently below 1.5% across the primary mechanical axes.

Tab. S5. Chemically recycled ABS (from Impressio ABS C06 counterpart P) Extrusion and Printing Parameters

| **PELLET PRE-DRYING** | |
| --- | --- |
| Temperature | 80°C for 2-4 hours |
| **FILAMENT EXTRUSION PARAMETERS** | |
| Temperature (°C) | 210 – 225 – 230 - 240 |
| Extrusion speed (rpm) | 4 |
| Filament thickness (mm) | 1,75 ± 0,005 |
| Cooling fan speed (%) | 15 |
| **PRINTING PARAMETERS** | |
| Nozzle temperature | 225 ± 260 |
| Bed temperature | 90 ± 110 |
| Flow | 98 ± 105 |
| Layer thickness (mm) | 0.2 |
| Printing speed (mm/s) | 30 ± 50 |
| Retraction (mm) | 0.8 ± 1.2 |

Fig. S13. Chemically recycled ABS Dog-Bone Type 1A

Fig. S14. Chemically recycled ABS Dog-Bone Type 1B

Fig. S15. Chemically recycled ABS Solid Cube, Flow Test, and Temp Tower
